# Supplementary material for: Immediate myeloid depot for SARS-CoV-2 in the human lung
Source: Res Sq. 2022 May 17:rs.3.rs-1639631. Preprint. [Version 1] doi: 10.21203/rs.3.rs-1639631/v1 (PMC9128787; doi:10.21203/rs.3.rs-1639631/v1)
Supplement: 1 [file NIHPPRS1639631V1-supplement-1.pdf]

Supplementary Table 1. Human lung donor demographics and experimental assignments.

Donor	Gender	Race/ Ethnicity	Age	Cause of death	Donation	Comorbidities	Smoking (>20PY)	Experiment Type
**1**	M	White	63	Anoxia	DBD	Hypertension, Pulmonary nodules	yes	BAL
**2**	F	White	49	Anoxia	DCD	Asthma, COPD, Sepsis pneumonia	yes	PCLS flow, PCLS imaging
**3**	F	White	37	Anoxia	DBD	None	no	PCLS flow, PCLS imaging
**4**	M	White	51	Stroke	DBD	Diabetes, CAD, Hypertension	no	PCLS flow, PCLS imaging
**5**	M	White	47	Stroke	DCD	None	no	PCLS flow, PCLS imaging
**6**	M	Asian/White	32	Head Trauma	DBD	Diabetes	no	PCLS flow
**7**	F	Hispanic	44	Stroke	DBD	None	no	PCLS flow, PCLS imaging
**8**	M	Asian	50	Stroke	DCD	Asthma, Hypertension	no	PCLS flow
**9**	M	White	46	Suicide	DCD	None	yes	PCLS flow
**10**	F	Asian	56	Stroke	DCD	Hypertension	no	PCLS imaging
**11**	F	White	57	Stroke	DBD	None	no	PCLS flow, PCLS imaging
**12**	M	Hispanic	27	Head Trauma	DBD	Asthma	no	PCLS SCS
**13**	M	White	43	Anoxia	DCD	None	no	BAL, PCLS flow, PCLS imaging
**14**	F	Hispanic	44	Stroke	DBD	None	no	PCLS imaging, PCLS SCS
**15**	M	White	43	Anoxia	DCD	None	no	PCLS flow
**16**	M	Hispanic	57	Stroke	DBD	None	no	BAL
**17**	M	White	50	Head Trauma	DCD	Hypertension	no	BAL
**18**	M	White	31	Head Trauma	DCD	None	no	BAL
**19**	M	Hispanic	50	Stroke	DBD	Hypertension	no	BAL
**20**	F	White	62	Head Trauma	DBD	Cancer	no	BAL
**21**	M	White	27	Anoxia	DBD	None	no	BAL
**22**	F	Hispanic	39	Head trauma	DBD	None	no	BAL
**23**	F	White	58	Anoxia	DCD	Diabetes, Hypertension	no	BAL
**24**	F	White	62	Anoxia	DCD	COPD, Hypertension, Hypersensitivity Lung Disease, and diabetes	yes	BAL
**25**	M	White	32	Anoxia	DCD	Asthma	no	BAL
**26**	M	Hispanic	25	Anoxia	DCD	None	no	BAL
**27**	M	Hispanic	33	Head trauma	DBD	Hypertension	no	BAL
**28**	M	White	33	Anoxia	DBD	Diabetes	yes	BAL
**29**	M	White	46	Stroke	DBD	Asthma	no	BAL

DBD: donation after brainstem death
COPD: chronic obstructive pulmonary disease
BAL: bronchoalveolar lavage
PCLS Imaging: precision cut lung slice immunofluorescence imaging
DCD: donation after cardiac death
CAD: coronary artery disease
PCLS flow: precision cut lung slice flow cytometry
SCS: single-cell RNA sequencing

Supplementary Table 2. Demographic and clinical information for COVID-19 ETA samples.

Subject	Age	Gender	Ethnicity	ARDS (Y/N) Berlin definition	ETA sampling after intubation (days)	P/F ratio at time of ETT sampling	ICU LOS (days)	Hospital LOS (days)	Death (Y/N)
1 (1)	34	F	White	Yes	0	180	12	14	No
2 (365)	55	M	White	Yes	40	110	35	60	No
3 (414)	58	M	Other / Multiple Races	Yes	1, 3	165	19	27	No
4 (415)	62	F	Black / African American	Yes	2	124	14	21	No
5 (419)	68	M	Other / Multiple Races	Yes	7	175	26	50	No
6 (476)	59	M	White	Yes	12	160	44	78	No
7 (389)	66	F	White	Yes	2	144	39	55	No

ARDS: acute respiratory distress syndrome
ETA: endotracheal tube aspirate
P/F ratio: PaO_2/FiO_2
ICU LOS: intensive care unit length of stay
Hospital LOS: hospital length of stay

Supplementary Table 3. Flow cytometry panel for PCLS experiments.

Staining	Antibody (clone)	Lot number	Dye	Catalog number (Supplier)
Surface Staining	CD169	1621398	AF594	FAB5197T (R&D Systems)
	ACE2	BJ07067522	PE	bs-1004R (BIOSS)
	EpCAM	B250368	BV650	324226 (BioLegend)
	CD31 (WM59)	8232937	BV605	562855 (BD Biosciences)
	CD45 (HI30)	B333796	BV421	304032 (BioLegend)
	CD14 (M5E2)	B275828	BV711	301838 (BioLegend)
	CD3 (SK7)	0314461	BB700	566575 (BD Biosciences)
	CD19 (SJ25C1)	1069967	BB700	566396 (BD Biosciences)
	Zombie (viability)	B331984	NIR	77184 (BioLegend)
	HLA-DR (G46-6)	1266420	BUV395	564040 (BD Biosciences)
Intracellular Staining	Spike	1619059	AF647	FAB105805R (R&D Systems)
	dsRNA (J2)	J2-2007	AF488	10010 (Scicons)

Supplementary Table 4. Flow cytometry panel for BAL experiments.

Staining	Antibody	Lot number	Dye	Reference
**Surface Staining**	CD169	1621398	AF594	FAB5197T (R&D Systems)
	ACE2	BJ07067522	PE	bs-1004R (BIOSS)
	CD15 (W6D3)	1089776	BV786	741013 (BD Biosciences)
	CD16 (3G8)	B321940	BV605	302040 (BioLegend)
	CD45 (HI30)	B333796	BV421	304032 (BioLegend)
	CD14 (M5E2)	B275828	BV711	301838 (BioLegend)
	CD3 (SK7)	0314461	BB700	566575 (BD Biosciences)
	CD19 (SJ25C1)	1069967	BB700	566396 (BD Biosciences)
	Zombie (viability)	B331984	NIR	77184 (BioLegend)
	HLA-DR (G46-6)	1266420	BUV395	564040 (BD Biosciences)
**Intracellular Staining**	Spike	1619059	AF647	FAB105805R (R&D Systems)
	IFITM3 (EPR5242)	GR3416716-1	AF488	Ab198559 (Abcam)
